# HLA-mismatched allogeneic adoptive immune therapy in severely immunosuppressed AIDS patients

**DOI:** 10.1038/s41392-021-00550-2

**Published:** 2021-05-07

**Authors:** Ruonan Xu, Ji-Yuan Zhang, Bo Tu, Zhe Xu, Hui-Huang Huang, Lei Huang, Yan-Mei Jiao, Tao Yang, Chao Zhang, En-Qiang Qin, Tian-Jun Jiang, Yun-Bo Xie, Yuan-Yuan Li, Lei Jin, Chun-Bao Zhou, Ming Shi, Mei Guo, Hui-Sheng Ai, Linqi Zhang, Fu-Sheng Wang

**Affiliations:** 1 Treatment and Research Center for Infectious Diseases, The Fifth Medical Center, PLA General Hospital, Beijing, China; 2 Department of Hematology and Transplantation, The Fifth Medical Center, PLA General Hospital, Beijing, China; 3 Comprehensive AIDS Research Center, School of Medicine, Tsinghua University, Beijing, China

**Keywords:** Infectious diseases, Translational research

## Abstract

Severely immunosuppressed AIDS patients with recurrent opportunistic infections (OIs) represent an unmet medical need even in the era of antiretroviral therapy (ART). Here we report the development of a human leukocyte antigen (HLA)-mismatched allogeneic adaptive immune therapy (AAIT) for severely immunosuppressed AIDS patients. Twelve severely immunosuppressed AIDS patients with severe OIs were enrolled in this single-arm study. Qualified donors received subcutaneous recombinant granulocyte-colony-stimulating factor twice daily for 4–5 days to stimulate hematopoiesis. Peripheral blood mononuclear cells were collected from these donors via leukapheresis and transfused into the coupled patients. Clinical, immunological, and virological parameters were monitored during a 12-month follow-up period. We found AAIT combined with ART was safe and well-tolerated at the examined doses and transfusion regimen in all 12 patients. Improvements in clinical symptoms were evident throughout the study period. All patients exhibited a steady increase of peripheral CD4^+^ T cells from a median 10.5 to 207.5 cells/μl. Rapid increase in peripheral CD8^+^ T-cell count from a median 416.5 to 1206.5 cells/μl was found in the first 90 days since initiation of AAIT. In addition, their inflammatory cytokine levels and HIV RNA viral load decreased. A short-term microchimerism with donor cells was found. There were no adverse events associated with graft-versus-host disease throughout the study period. Overall, AAIT treatment was safe, and might help severely immunosuppressed AIDS patients to achieve a better immune restoration. A further clinical trial with control is necessary to confirm the efficacy of AAIT medication.

## Introduction

Chronic human immunodeficiency virus (HIV)-1 replication leads to progressive loss of CD4^+^ T cells and immune disorders. Thus, acquired immune deficiency syndrome (AIDS) induced by chronic HIV-1 infection is not only a viral disease, but also an immunological illness. Combined antiretroviral therapy (ART) efficiently suppresses viral replication and promotes an increase in CD4^+^ T cells, thereby reducing the mortality of AIDS patients, however it fails to efficiently resolve the immunological issues.^1^ As a result, AIDS patients undergoing ART still suffer from non-AIDS-related events associated with systemic immune-mediated inflammation,^2^ and are often with low CD4^+^ T-cell count even after 2 years of efficient ART.^3^ Furthermore, chronic HIV-1 infection can severely destroy the immune system, leading to extremely low CD4^+^ T-cell counts <50 cells/μl. Such severely immunosuppressed AIDS patients are often diagnosed at late stage and suffer from intractable opportunistic infections (OIs), severe wasting syndromes, and other serious complications like immune response inflammation syndrome (IRIS).^4–6^

Due to the complication of OIs, some severely immunosuppressed AIDS patients cannot tolerate ART and have a higher likelihood of premature death. In last decade, more than 5% newly diagnosed HIV-1 cases with baseline CD4^+^ T-cell counts <50 cells/μl died in the first year in China.^7^ Therefore, even in the era of ART, more effective immune interventions are required for advanced AIDS patients, especially for severely immunosuppressed ones.

Complementary immune therapy against AIDS disease has been used since the early 1980s. For example, HLA-matched allogeneic lymphocyte transfusion was used to treat AIDS patients either alone or in combination with bone marrow transplantation.^8–11^ However, these early strategies were hampered by the lack of efficient antiretroviral agents and thus only resulted in a transient elevation in peripheral immunocyte microchimerism within 2–4 weeks. With the advent of ART in the 1990s, the survival rate greatly raised, particularly among AIDS patients with leukemia or lymphoma, primarily due to autologous or HLA-matched allogeneic hematopoietic stem cell transfusion (HSCT). Unfortunately, allogeneic HSCT is limited to AIDS patients with hematopoietic malignancies.^12^ The most successful examples are the “Berlin patient” and “London patient,” who received both ART and homozygous CCR5Δ32 allogeneic HSCT, finally achieved functional cure for AIDS and remission of their hematopoietic malignancies.^13,14^ However, given the rarity of HLA-identical donors with a CCR5Δ32 homozygous genotype and associated life-threatening adversaries, such success is not replicable to the vast majority of patients in the routine clinical setting.

Recently, efforts have been made to explore genetically modified CD4^+^ T cells and HSCs from autologous or HLA-matched donors to restore the immune response in HIV-1-infected patients. Participating patients are generally required to have a good response to ART and reasonable levels of CD4^+^ T-cell counts (usually >200 cells/μl).^15–17^ However, few treatment strategies are designed to treat severely immunosuppressed AIDS patients with CD4^+^ T-cell counts <50 cells/μl.

To address these issues, we developed a HLA-mismatched allogeneic adoptive immune therapy (AAIT) combined with ART regimen specifically tailored for severely immunosuppressed AIDS patients with CD4^+^ T-cell counts <50 cells/μl. Here we present the safety and preliminary efficacy of this combined therapy.

## Results

### Baseline characteristics of enrolled patients

**Fig. 1:**
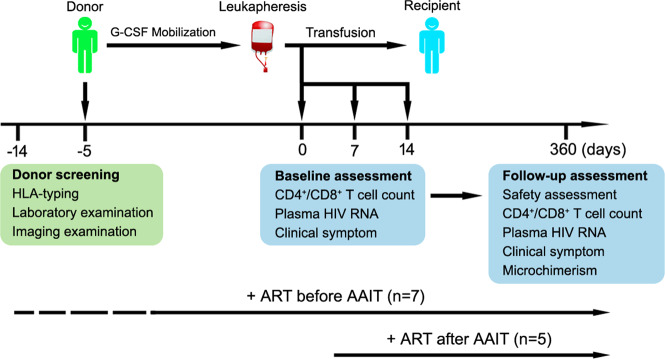
Structure of the phase I, single-arm, AAIT study. AAIT was performed in 12 severely immunosuppressed AIDS patients. Seven patients received AAIT after ART, and five received AAIT before ART. Anti-OI treatments were given as necessary during the immunotherapy. G-CSF granulocyte-colony-stimulating factor, AAIT allogeneic adoptive immune therapy, OIs opportunistic infections, ART antiretroviral therapy

A total of 12 severely immunosuppressed AIDS patients were enrolled in this proof-of-concept study (Fig. 1). The demographic and baseline information of these patients at enrollment is summarized in Table 1. All patients had CD4^+^ T-cell counts well below 33 cells/μl, and 10 patients had counts <20 cells/μl. All patients presented two to five serious OIs, including pneumocystis carinii pneumonia, cryptosporidiosis, oral candidiasis, etc. Some patients had detectable cytomegalovirus (CMV) or Epstein–Barr virus (EBV) nucleic acid in the peripheral blood. No hepatitis B virus (HBV) or hepatitis C virus (HCV) nucleic acid was detected in any of the patients.

**Table 1:** Baseline characteristics of 12 severely immunosuppressed AIDS patients enrolled in AAIT

Patient	Gender	Age (years)	Transmission route	Laboratory examinations										Viral nucleic acid				ART relative to initiation of AAIT (days)	Times of transfusion	Dose of each transfusion 10^8^/kg
				HIV RNA (copies/ml)	CD4 (cells/μl)	CD8 (cells/μl)	WBC (10^9^/L)	RBC (10^12^/L)	PLT (10^9^/L)	HGB (g/L)	ALT (U/L)	AST (U/L)	Cre (μmol/L)	CMV (IU/ml)	EBV (IU/ml)	HBV (copies/ml)	HCV (copies/ml)			
01-W	M	36	Heterosexual	<20	33	602	5.43	3.37	352	105	9	18	81	<100	2.58E+02	<40	<50	−104	5	3/2.5/2.5/ 2.5/2.5
02-Z	M	28	Heterosexual	2.17E+04	7	227	1.31	2.59	136	78	20	42	53	<100	1.97E+02	<40	<50	−28	4	3/3.1/3/3
03-G	M	18	Homosexual	2.32E+06	9	688	3.15	4.18	131	121	58	35	74	<100	<100	<40	<50	−5	3	3/3.5/2.6
04-D	M	35	Homosexual	1.62E+02	1	40	1.75	3.05	170	87	18	24	63	4.17E+02	<100	<40	<50	−58	3	2.6/2.9/2.9
05-Z	M	29	Homosexual	8.88E+05	32	778	1.88	4.41	142	127	95	68	88	<100	<100	<40	<50	+20	3	3/2.6/2.6
06-L	M	44	Homosexual	1.01E+06	11	90	6.25	4.38	394	128	40	38	62	1.74E+04	6.64E+02	<40	<50	−10	2	3/2.5
07-X	M	34	Unknown	1.76E+07	4	590	3.16	4.44	239	127	29	46	84	<100	<100	<40	<50	−6	3	3.5/3/3
08-D	M	54	Homosexual	2.20E+05	10	268	7.09	4.66	325	151	33	18	85	<100	<100	<40	<50	+8	2	3/3
09-L	F	31	Heterosexual	1.25E+07	13	474	4.27	3.96	87	127	94	98	47	1.27E+03	<100	<40	<50	+2	3	3.3/3.2/3.2
10-L	M	35	Heterosexual	4.47E+05	20	374	4.32	3.76	278	121	47	27	74	<100	<100	<40	<50	+7	2	3.5/2.6
11-S	M	39	Unknown	2.53E+05	7	258	2.29	5.83	281	165	59	39	69	<100	<100	<40	<50	−3	2	3.1/3
12-L	M	27	Homosexual	8.86E+04	15	459	2.87	4.63	106	133	112	32	72	3.04E+04	<100	<40	<50	+2	2	3.3/3

“−” means ART before AAIT treatment, “+” means ART after AAIT treatment

*M* male, *F* female, *WBC* white blood cell, *RBC* red blood cell, *PLT* platelet, *HGB* hemoglobin, *ALT* alanine aminotransferase, *AST* glutamic oxaloacetic transaminase, *Cre* creatinine, *CMV* cytomegalovirus, *EBV* Epstein–Barr virus, *HBV* hepatitis B virus, *HCV* hepatitis C virus

The majority of patients also presented with systemic constitutional symptoms such as intermittent or continuous fever (38 °C) for more than 1 month, repeated night sweats, debilitating fatigue, persistent diarrhea, and loss of body weight (Table 2). The plasma viral load ranged from undetectable to 1.76 × 10^7^ copies/ml.

**Table 2:** AAIT improved clinical events and symptoms in the treated 12 patients

Patient	Symptoms before AAIT		Clinical response after AAIT treatment
	AIDS-related events at enrollment	Systemic constitutional symptoms at enrollment	
01-W	Pneumonia Sepsis Crysptosporidiosis EBV infection Wasting syndrome	+++	No new opportunistic infections, doing well
02-Z	Pneumonia Pulmonary abscess Colitis EBV infection Wasting syndrome	+++++	Pulmonary abscess significantly improved within 1 month, doing well
03-G	Pneumonia PCP	++	No new opportunistic infections, doing well
04-D	Pneumonia CMV infection Wasting syndrome	++++	No new opportunistic infections, doing well
05-Z	Pneumonia PCP Oral candidiasis	++	No new opportunistic infections, doing well
06-L	Pneumonia PCP CMV infection EBV infection	++	No new opportunistic infections, doing well
07-X	Pneumonia PCP Oral candidiasis	+++	Phlegmon occurs 37 days later
08-D	Pneumonia PCP Oral candidiasis Wasting syndrome	+++	No new opportunistic infections, doing well
09-L	Cryptosporidium enteritis Salmonella typhimurium enteritis Oral candidiasis CMV infection Wasting syndrome	++++	No new opportunistic infections, doing well
10-L	Pneumonia PCP	++	No new opportunistic infections, doing well
11-S	Pneumonia PCP Oral candidiasis	++	No new opportunistic infections, doing well
12-L	Pneumonia PCP Oral candidiasis CMV infection	++	No new opportunistic infections, doing well

Clinical events and symptoms of 12 severely immunosuppressed AIDS patients were carefully recorded within 1 year after AAIT treatment. Systemic constitutional symptoms included intermittent or continuous fever (38 °C) for more than 1 month repeated night sweats, debilitating fatigue, persistent diarrhea, and a loss of body weight of more than 10%, each “+” represents a symptom

*PCP* pneumocystis carinii pneumonia, *EBV* Epstein–Barr virus, *CMV* cytomegalovirus

Anti-OI treatments were initiated according to current guidelines.^18^ Seven patients received the first-line ART regimen before AAIT; the remaining five patients received ART after AAIT, depending on their tolerance to the ART regimen.

### AAIT is safe and well-tolerated

We selected 12 HLA-mismatched healthy donors and one HLA-matched donor from the relatives of the 12 patients (Supplementary Table 1) through determining their HLA haplotype on HLA-A, B, C; DRB1; and DQB1 alleles. After treatment with subcutaneous injection of recombinant human granulocyte-colony-stimulating factor (G-CSF) at 5 μg/kg twice daily for 4–5 consecutive days, when the total number of peripheral lymphocytes and monocytes exceeded 4.5 × 10^9^ cells/L, donors were leukapherased to collect the G-CSF mobilized mononuclear cells (G-MNCs). Freshly collected mononuclear cells at an average dose of 3 × 10^8^ cells/kg (range 2.5–3.5 × 10^8^ cells/kg) were transfused into the recipient on day 0, while the remaining G-MNCs were aliquoted and stored in liquid nitrogen for later use. All patients received consecutive transfusions on day 7 and 14. Up to five rounds of transfusions were performed for some participating patients, depending on the need of the recipients and the availability of G-MNCs (Table 1).

G-CSF transfusion resulted in a significantly increased white blood (median value increased from 5.97 × 10^9^/L to 42.15 × 10^9^/L) and neutrophil cell (median value increased from 3.60 × 10^9^/L to 35.85 × 10^9^/L) in all healthy donors; a similar trend was observed for lymphocytes (median value increased from 1.65 × 10^9^/L to 3.33 × 10^9^/L) and monocytes (median value increased from 0.30 × 10^9^/L to 2.11 × 10^9^/L), albeit to a lesser degree. The components of infused G-MNCs were listed in Supplementary Table 2 in detail.

Over the 12-month follow-up period, AAIT was safe and well-tolerated at the examined doses and transfusion regimens in all 12 patients. The criteria for monitoring adverse events (AEs) were accorded to Common Terminology Criteria for Adverse Events (CTCAE), any abnormalities after transfusion were carefully recorded. None of the patients experienced IRIS and only two had transient transfusion-related fever in the first 72 h after initiation of AAIT, which recovered quickly.

Improvements in clinical symptoms were evident throughout the study period, particularly in controlling the high fever, chronic diarrhea, weight loss, and serious pneumonia. For example, two patients (01-W and 02-Z) had persistent high fever, high frequency of diarrhea, and serious pneumonia before AAIT, these symptoms were significantly improved within 2–4 weeks after treatment (Fig. S1). In addition, five patients with wasting syndrome (01-W, 02-Z, 04-D, 08-D, and 09-L) improved significantly after AAIT, with substantial increases in appetite and weight gain. AIDS-related events and unscheduled hospitalization due to serious OIs were not found during follow-up (Table 2). Last, there was no acute (within 100 days) or chronic (within 360 days) graft-versus-host disease (GVHD) that occurred in skin, mouth, gastrointestinal tract, liver, and lung (Table 3).^19,20^

**Table 3:** GVHD assessment of 12 severely immunosuppressed AIDS patients within 1 year after AAIT

Patient	Side effects	Clinical manifestations <100 days					100 days < Clinical manifestations < 360 days				
		Skin (Erythema maculopapular rash)	Mouth (Gingivitis mucositis	GI tract (Diarrhea anorexia)	Liver Total bilirubin(umol/L) ALT(U/L) AST(U/L)	Lung (Recurrent pneumonia)	Skin (Erythema maculopapular rash)	Mouth (Gingivitis mucositis)	GI tract (Diarrhea anorexia)	Liver Total bilirubin (umol/L) ALT(U/L) AST(U/L)	Lung (Recurrent pneumonia)
01-W	Transient fever within 72 h	No	No	No	Normal	No	No	No	No	Normal	No
02-Z	No	No	No	No serious diarrhea 14 days and no anorexia 3 days since first time of AAIT	Normal	No	No	No	No	Normal	No
03-G	No	No	No	No	Normal	No	No	No	No	Normal	No
04-D	No	No	No	No	Normal	No	No	No	No	Normal	No
05-Z	No	No	HIV-associated herpes	No	ALT at double level at baseline, decreased to normal within 1 month	No	No	No	No	Normal	No
06-L	No	No	No	No	Normal	No	No	No	No	Normal	No
07-X	No	ART-induced rash	HIV-associated oral candidiasis	No	Normal	No	No	No	No	Normal	No
08-D	No	No	HIV-associated oral candidiasis	No	Normal	No	No	No	No	Normal	No
09-L	Transient fever within 48 h	No	HIV-associated oral candidiasis	No diarrhea 10 days since first time of AAIT	ALT at double level at baseline, decreased to normal within 1 week	No	No	No	No	Normal	No
10-L	No	No	No	No	Normal	No	No	No	No	Normal	No
11-S	No	No	HIV-associated oral candidiasis	No	Normal	No	No	No	No	Normal	No
12-L	No	No	HIV-associated oral candidiasis	No	Normal	No	No	No	No	Normal	No

Clinical changes in the skin, mouth, gastrointestinal tract, and liver were carefully checked 1 year after AAIT

*GI* gastrointestinal, *ALT* alanine aminotransferase, *AST* aspartate aminotransferase, *AAIT* allogeneic adoptive immune therapy

The levels of inflammatory cytokines were screened at days 0, 30 and day 360 after AAIT. There was no significant increase of IL-6 and IL-8, which were related to cytokine-release syndrome.^21^ Levels of IL-2, IL-4, IL-10, IL-17A, IFN-r, IP-10, CRP, MCP-1, GM-CSF, and sCD163 decreased over the 12-month study period, obviously within the first 30 days (Fig. S2).

### AAIT facilitates immune restoration and viral suppression

**Fig. 2:**
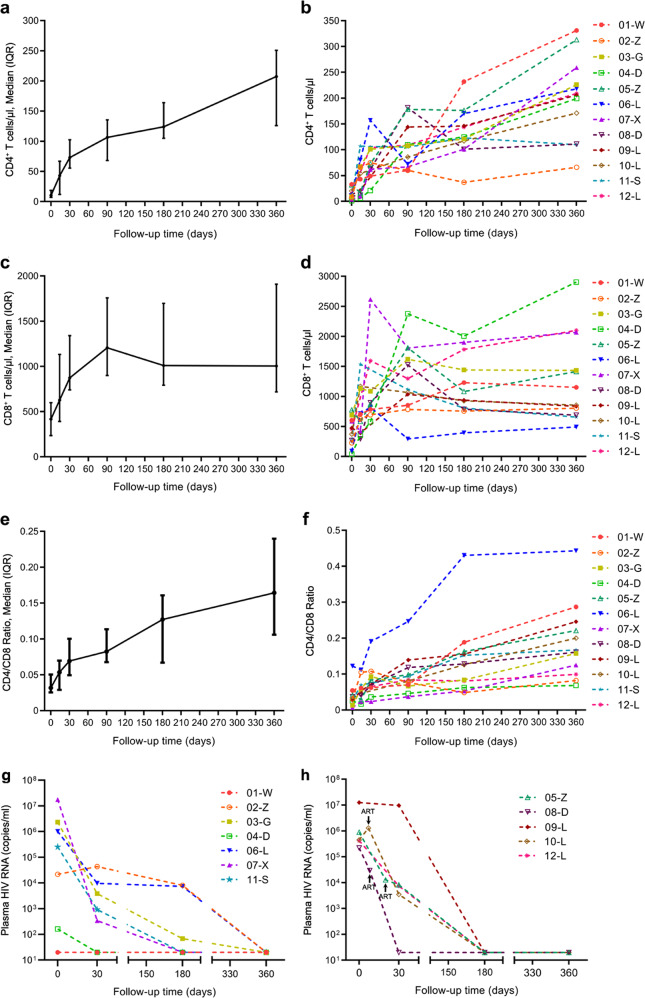
Effects of AAIT on peripheral blood CD4+/CD8+ T-cell counts and HIV RNA in severely immunosuppressed AIDS patients. **a** The trend of CD4^+^ T-cell counts in severely immunosuppressed AIDS patients. **b** Changes in CD4^+^ T-cell counts in each of the 12 severely immunosuppressed AIDS patients. **c** The trend of CD8^+^ T-cell counts in severely immunosuppressed AIDS patients. **d** Changes in CD8^+^ T-cell counts in each of the 12 severely immunosuppressed AIDS patients. **e** The trend of CD4/CD8 ratio in severely immunosuppressed AIDS patients. **f** Changes in CD4/CD8 ratio in each of the 12 severely immunosuppressed AIDS patients. **g** The change of HIV RNA in patients receiving ART before AAIT (*n* = 7). **h** The change of HIV RNA in patients receiving AAIT before ART (*n* = 5). For patient 05-Z, the AAIT was 20 days before ART. For patient 08-D, the AAIT was 8 days before ART. For patient 10-L, the AAIT was 7 days before ART. For the other two patients, the AAIT was 2 days before ART. The arrows represent the time of ART start

During the 12-month follow-up period, we closely monitored the dynamic changes in CD4^+^ and CD8^+^ T-cell counts and plasma viral load. Median CD4^+^ T-cell counts increased from 10.5 to 207.5 cells/μl (Fig. 2a, b), while median CD8^+^ T-cell counts increased from 416.5 to 1004 cells/μl (Fig. 2c, d). In addition, the median CD4/CD8 ratio raised from 0.032 to 0.164 (Fig. 2e, f).

To identify the source of these increased cells, we performed a microchimerism assay on the peripheral blood mononuclear cells up to 45 days after AAIT initiation. In most of the cases, microchimerisms peaked on day 1, and then dropped suddenly, becoming undetectable by day 7 (Fig. S3). In one patient (02-Z) who received two rounds of transfusions, separately from a HLA-matched donor and a HLA-mismatched donor, the changes in microchimerisms appeared to be similar (i.e., peaking on day 1, and then rapidly reducing in the ensuing days). However, compared to the HLA-matched infusion, the levels of microchimerisms were lower in the HLA-mismatched transfusion.

A significant drop in plasma viral load was found in all patients following the initiation of AAIT and ART. Two patients (02-Z and 06-L) switched ART treatment regimen from 3TC/TDF/EFV to 3TC/AZT/DTG or 3TC/AZT/LPV/r, respectively, due to drug resistance. Another two patients (01-W and 08-D) changed the regimen from 3TC/TDF/EFV to 3TC/TDF/LPV/r or 3TC/TDF/NVP, respectively, due to intolerance to EFV (Supplementary Table 3). Interestingly, AAIT appeared to exert antiretroviral activity, even without ART. For instance, patients 05-Z and 08-D, who separately received ART 8 or 20 days after AAIT treatment, demonstrated more than one log decline in plasma viral load (Fig. 2h). Patient 10-L showed a slight increase in plasma viral load during the first 7 days after initiation of AAIT, but the viral load subsequently declined to undetectable level after 180 days. With these results, AAIT showed good safety and tolerability, as well as the ability to facilitate immune restoration and viral suppression in patients with CD4^+^ cell counts <50 cells/μl of blood.

## Discussion

There have been no safe and effective immune intervention that can be used alongside ART to address the unmet medical need for advanced AIDS patients, especially those severely immunosuppressed. We have successfully developed AAIT as an innovative regimen consisting of four successive procedures: (1) screening and selection of healthy donors; (2) mobilization with G-CSF; (3) G-MNCs leukapheresis; and (4) G-MNCs transfusion. We have demonstrated that HLA-mismatched AAIT is not only safe and tolerable, but also contribute to improve the clinical symptoms as well as key immunological and virological parameters associated with AIDS-related events during this 12-month study. Therefore, AAIT represents a promising treatment strategy for immune restoration and virological suppression in advanced AIDS patients.

AAIT demonstrated impressive safety, tolerability, and feasibility, and contributed to rapid control of OIs. No serious AEs and no GVHD response were found in any patients throughout the study period, and no cytokine response storm associated with AAIT occurred. Among AAIT-treated patients, the common systemic symptoms including intermittent or persistent fever, repeated night sweats, debilitating fatigue, refractory diarrhea, and loss of body weight were significantly improved or completely disappeared; notably, the appetite and body weight were significantly increased. Through adoptive transferring a large number of neutrophils, CD4^+^ T and CD8^+^ T lymphocytes, natural killer (NK) cells, and monocytes from healthy donors, AAIT medication might reshape the whole immune environment and enhance the innate and adaptive immunity. In particular, AAIT in combination with ART may jointly contribute to a sustained increase of CD4^+^ T-cell count and the clinical benefits as mentioned above.

We also showed that AAIT facilitates both immune restoration and viral suppression. During the 1-year follow-up period, we found a substantial and steady increase in both CD4^+^ and CD8^+^ T-cell counts accompanied by a significant drop in plasma viral load. But no transfusion-associated immune reconstitution inflammatory syndrome occurred in any of the patients owning to the swiftly recover of CD4^+^ T-cell count. Inflammatory-mediated cytokine levels also decreased within 30 days of AAIT. Moreover, a transient microchimerism with donor cells was found in recipients. Although the exact mechanism underlying immune restoration remains to be determined, the transient and low percentage of microchimerism in the recipients suggests the increased CD4^+^ and CD8^+^ T cells likely originate from the recipients’ own cells. Interestingly, the microchimerism levels are higher and persist longer when HLA-matched donors are used compared to mismatched donors. Microchimerism levels also tended to decrease after repeated G-MNC transfusions. These results support the hypothesis that the host-versus-graft effect contributes to the immune restoration in the recipients. However, not every patient in our cohort demonstrated an improved CD4^+^ T-cell count and the underlying reasons are currently unclear.

Our results also indicate that AAIT exerts antiretroviral activity, even without ART. Two patients who received ART after AAIT demonstrated a more than one log decline in plasma viral load. It is possible that infused CD8^+^ T cells, NK cells, or some other cell types exercise some nonspecific antiviral functions through modulating the patient’s immune system. This finding is consistent with previous studies in which allogeneic treatment exerted a vigorous graft-versus-virus effect.^22,23^

Notably, our AAIT protocol does not use preconditioning, unlike the regimens used for hematopoietic malignant patients.^11,12^ This is primarily due to the unique clinical setting of severely immunosuppressed AIDS recipients who have almost complete eradication of adaptive and innate immunities, and lose the ability to reject the transfused allogenic G-MNCs from the donors. However, the underlying mechanisms need to be further studied.

There are some limitations in our proof-of-concept study. First, small cases were included and lacked a control group to determine whether combined AAIT/ART is better than ART monotherapy. Second, all patients were simultaneously accepted anti-OIs therapy, so it is unclear if these patients would have quickly recovered without the AAIT. Third, we also found that some AAIT-treated patients did not show improved CD4^+^ T-cell counts. Whether the efficacy of AAIT is influenced by the recipients’ immune environment, their HLA status, or other reasons, requires further investigation.

In conclusion, we have developed a feasible and effective therapeutic regimen, named AAIT, for severely immunosuppressed AIDS patients. Our findings indicated that HLA-mismatched AAIT is well-tolerated, and may contribute to restoration of the immune system in severely immunosuppressed AIDS patients. Following this phase 1 study, we have now embarked on a multi-center, open-labeled, phase 2 nonrandomized trial to assess the safety and efficacy of AAIT for the treatment of advanced AIDS patients (ClinicalTrials.gov Identifier: NCT 04098770).

## Methods and materials

### Study patients

A total of 12 severely immunosuppressed AIDS patients were enrolled in this single-armed clinical study from September 2015 to August 2017. The inclusion criteria were: (1) age 18–60 years, (2) severe immunodeficiency with HIV-1 infection and peripheral CD4^+^ T-cell counts <50 cells/μl, and (3) evidence of serious AIDS-related complications. Patients with the following features were excluded: (1) tumor and other serious organ diseases unrelated to AIDS, (2) allergy to blood products, or (3) under long-term immunosuppressive therapy. The baseline characteristics of the enrolled patients are listed (Table 1). Our study was approved by the ethics board of the Fifth Medical Center of the Chinese PLA General Hospital and all participating patients provided informed consent.

### Development of the AAIT protocol

The AAIT protocol included four successive procedures: (1) screening and selection of healthy donors; (2) G-CSF mobilization; (3) G-MNCs leukapheresis; and (4) G-MNCs transfusion (Fig. 1). Healthy donors were selected from HLA-mismatched relatives of the enrolled AIDS patients through determining their HLA haplotype on HLA-A, B, C; DRB1; and DQB1 alleles.^24–26^ Physical examinations and laboratory tests were conducted for each donor, as previously described.^24,25^ Donors with virus infection (including HIV, hepatitis A virus, HBV, HCV, hepatitis E virus, EBV, CMV, and treponema pallidum) were excluded.

Qualified donors were treated with a subcutaneous injection of 5 μg/kg of rhG-CSF (HKyowa Hakko Kirin Co., Ltd), twice a day for 4–5 consecutive days. When the total number of peripheral lymphocytes and monocytes in the donors exceeded 4.5 × 10^9^ cells/L, G-MNCs were obtained through leukapheresis by using a cell separator (COM.TEC, Fresenius Kabi), and the components of the G-MNCs were analyzed by FACS analysis, as previously described.^24,25^ Fresh G-MNCs were used for the first transfusion in recipients individually on day 0, and the remaining G-MNCs were divided into aliquots and stored in liquid nitrogen. On days 7 and 14, about 3 × 10^8^ cells/kg (range, 2.5–3.5 × 10^8^ cells/kg) of mononuclear cells were transfused. Based on the need of the recipients and the availability of G-MNCs, up to five rounds of transfusions were performed for some patients. The time and dose of each of the G-MNCs transfusions for each patient are summarized in Table 1.

### Safety and efficacy assessments

The primary endpoint of our study was the safety assessment. Secondary endpoints focused on clinical, immunological, and virological improvement. The follow-up period after AAIT was 12 months for all participating patients. Clinical safety was assessed by physical and experimental examinations, the criteria for monitoring AEs were accorded to CTCAE. GVHD-associated symptoms were monitored throughout the entire study period, as previously described.^27^ Peripheral CD4^+^ and CD8^+^ T-cell counts, plasma HIV-1 RNA levels, cytokine profiles, the occurrence of OIs, and AIDS-related events were recorded during the entire study period. An independent safety monitoring committee reviewed the data and recorded all AEs during the study.

### Monitoring of plasma HIV-1 RNA levels, and peripheral CD4^+^ and CD8^+^ T-cell counts

COBAS AmpliPrep was used to quantify HIV-1 RNA levels in the plasma samples, as previously described. Trucount Tubes (BD Biosciences) were used to detect CD4^+^ and CD8^+^ T-cell counts in the peripheral blood.^28^

### Detection of cytokine profiles

Serum samples obtained before and after AAIT treatment were analyzed separately, cytokine profiles (IL-2, IL-4, IL-6, IL-8, IL-10, IL-17A, IFN-r, IP-10, MCP-1, and GM-CSF) were detected by using the Luminex Bio-Plex Pro Human cytokine assay with the Luminex 200 system. CRP and sCD163 were detected by the ELISA kit (R&D Systems) according to the manufacturer’s protocols at days 0, 30, and 360.

### Detection of the components of the G-MNCs by FACS array

The components of the G-MNCs were stained by surface immunostaining with anti-CD3, CD34, CD45, CD4, CD8, CD19, CD16, CD56, CD25, and CD127 (BD PharMingen) antibodies, and analyzed using FACS Verse and FlowJo software (Tristar, San Carlos, CA), as previously described.^24^

### Detection of microchimerism

Microchimerism was analyzed by real-time quantitative PCR for the detection of indel genomic polymorphisms in the peripheral blood samples with a sensitivity of 0·025% at different follow-up time points.^29^ Suitable DNA sequences, which were present in the donor and absent in the recipient, were selected.

### Statistical analysis

This was a phase I, single-arm, proof-of-concept study. The data presented here represent the interquartile range of CD4^+^ T and CD8^+^ T-cell counts. The study was registered at ClinicalTrail.gov with number NCT02651376.

## Supplementary information

Supplementary manuscript

## Data Availability

All of the data generated and analyzed during this study are included in our manuscript.
