# Supplementary material for: HLA-mismatched allogeneic adoptive immune therapy in severely immunosuppressed AIDS patients
Source: Signal Transduct Target Ther. 2021 May 7;6:174. doi: 10.1038/s41392-021-00550-2 (PMC8102474; doi:10.1038/s41392-021-00550-2)
Supplement: Supplementary file 1 — Supplementary manuscript [file 41392_2021_550_MOESM1_ESM.docx]

**Supplementary information for**

**HLA-mismatched allogeneic adoptive immune therapy in severely immunosuppressed AIDS patients**

Ruonan Xu ^#1^, Ji-Yuan Zhang ^#1^, Bo Tu ^#1^, Zhe Xu ^#1^, Hui-Huang Huang ^1^, Lei Huang ^1^, Yan-Mei Jiao ^1^, Tao Yang ^1^, Chao Zhang ^1^, En-Qiang Qin ^1^, Tian-Jun Jiang ^1^, Yun-Bo Xie ^1^, Yuan-Yuan Li ^1^, Lei Jin ^1^, Chun-Bao Zhou ^1^, Ming Shi ^1^, Mei Guo ^2^, Hui-Sheng Ai ^2^, Linqi Zhang ^3^, Fu-Sheng Wang ^1^.

# These authors contributed equally to this work

Fu-Sheng Wang, MD ([fswang302@163.com](mailto:fswang302@163.com)), Treatment and Research Center for Infectious Diseases, The Fifth Medical Center, PLA General Hospital, Beijing, China.

This PDF file includes:

Table S1 to S3

Figure S1 to S3

**Supporting Results**

Supplementary Table 1. HLA status of the 12 severely immunosuppressed AIDS patients and related donors.

Supplementary Table 2. Details of blood cell counts before G-CSF mobilization and at leukopheresis, and the components of the G-MNCs collected by leukopheresis in donors.

Supplementary Figure 1. Effects of AAIT on clinical symptoms in severely immunosuppressed AIDS patients.

Supplementary Figure 2. Serum cytokine levels within 12 months after AAIT in patients.

Supplementary Figure 3. Dynamics of donor cell microchimerism following AAIT treatment.

Supplementary Table 3. ART regimens for 12 severely immunosuppressed AIDS patients.

|  | **Table S 1.** HLA status of the 12 severely immunosuppressed AIDS recipients and related donors. | | | | | | | | | | | | | | |  |
| --- | --- | --- | --- | --- | --- | --- | --- | --- | --- | --- | --- | --- | --- | --- | --- | --- |
|  | **Donor** | **Donor** | | | | | | | **Relationship with recipient** | **Recipient** | | | | | **HLA- mismatched**  **loci** |  |
|  |  | Gender | Age (years) | HLA-A | HLA-B | HLA-C | HLA-DRB1 | HLA-DQB1 |  | HLA-A | HLA-B | HLA-C | HLA-DRB1 | HLA-DQB1 |  |  |
|  | 01-W  donor | F | 53 | 03:01  33:03 | 07:02  51:01 | 07:02  14:02 | 13:01  13:01 | 06:03  06:03 | Mother | 03:01  24:02 | 40:06  51:01 | 14:02  15:02 | 13:01  14:04 | 05:03  06:03 | 5/10 |  |
|  | 02-Z  donors | F | 31 | 02:01  11:01 | 15:18  54:01 | 01:02  08:01 | 08:02  14:54/01(14:01:01G) | 04:02  05:03 | Sister  (Donor 1) | 02:01  11:01 | 15:18  54:01 | 01:02  08:01 | 08:02  14:54/01(14:01:01G) | 04:02  05:03 | 0/10 |  |
|  |  | M | 45 | 02:05  11:01 | 15:22  54:01 | 01:02  12:03 | 08:04  14:54/01(14:01:01G) | 04:02  05:03 | Brother  (Donor 2) |  |  |  |  |  | 6/10 |  |
|  | 03-G  donor | F | 48 | 02:01  11:02 | 46:01  51:01 | 01:02  14:02 | 04:04  09:01 | 03:02  03:03 | Mother | 11:02  24:02 | 07:06  51:01 | 7:02  14:02 | 09:01  12:02 | 03:03  05:02 | 5/10 |  |
|  | 04-D  donor | F | 40 | 24:02  24:02 | 40:01  40:02 | 03:04  03:04 | 04:03  15:01 | 03:02  03:02 | Sister | 01:01  24:02 | 35:02  40:02 | 03:04  04:01 | 04:03  11:04 | 03:01  03:02 | 5/10 |  |
|  | 05-Z  donor | F | 50 | 02:07  31:01 | 15:01  46:01 | 01:02  03:03 | 09:01  15:01 | 03:03  06:02 | Mother | 02:07  11:01 | 27:04  46:01 | 01:02  03:04 | 09:01  12:02 | 03:01  03:03 | 5/10 |  |
|  | 06-L donor | F | 38 | 11:01  29:01 | 13:02  51:01 | 06:02  14:02 | 04:01  07:01 | 02:02  03:01 | Sister | 02:01  24:02 | 35:01  54:01 | 01:02  04:01 | 11:01  04:01 | 03:01  05:03 | 9/10 |  |
|  | 07-X donor | F | 35 | 02:01  33:03 | 35:03  44:03 | 04:01  14:03 | 13:02  15:01 | 06:02  06:09 | Sister | 24:02  33:03 | 44:03  54:01 | 01:02  14:03 | 13:02  15:01 | 06:02  06:09 | 3/10 |  |
|  | 08-D donor | M | 41 | 01:01  11:01 | 38:02  57:01 | 06:02  07:02 | 07:01  07:01 | 02:02  03:03 | Brother | 01:01  68:01 | 27:05  57:01 | 02:02  06:02 | 04:06  07:01 | 03:02  03:03 | 5/10 |  |
|  | 09-L donor | F | 49 | 02:01  24:08 | 35:01  51:01 | 04:01  15:02 | 14:05  15:02 | 05:01  05:03 | Mother | 02:01  02:01 | 35:01  40:06 | 04:01  08:01 | 09:01  15:02 | 03:03  05:01 | 5/10 |  |
|  | 10-L donor | F | 43 | 24:02  24:02 | 15:02  48:01 | 08:01  08:01 | 09:01  15:01 | 03:03  06:02 | Sister | 02:03  24:02 | 15:12  48:01 | 03:03  08:01 | 12:02  15:01 | 03:01  06:02 | 5/10 |  |
|  | 11-S donor | M | 42 | 03:01  30:01 | 15:18  37:01 | 06:02  08:01 | 03:01  15:01 | 02:01  06:02 | Brother | 03:01  03:01 | 15:18  35:01 | 04:01  08:01 | 01:01  15:01 | 05:01  06:02 | 5/10 |  |
|  | 12-L donor | M | 27 | 02:01  03:01 | 40:01  44:02 | 05:01  07:02 | 12:02  13:01 | 03:01  06:03 | Brother | 02:01  11:01 | 51:01  51:01 | 14:02  14:02 | 09:01  09:01 | 03:03  03:03 | 9/10 |  |
|  | *HLA* human leukocyte antigen, *M* male, *F* female. | | | | | | | | | | | | | | |  |

**Table S 1.** HLA status of the 12 severely immunosuppressed AIDS patients and related donors.

HLA haplotypes (HLA-A, B, C; DRB1; and DQB1 alleles) were carefully recorded for the 12 patients and their related donors. Ten alleles in HLA-A, B, C; DRB1; and DQB1 were analyzed, and differences in alleles were counted as HLA-mismatched loci. HLA, human leukocyte antigen.

|  | **Table S 2.** Details of blood cell counts before G-CSF mobilization and at leukopheresis, and the components of the G-MNCs collected by leukopheresis in donors. | | | | | | | | | | | | | | | | | |  |
| --- | --- | --- | --- | --- | --- | --- | --- | --- | --- | --- | --- | --- | --- | --- | --- | --- | --- | --- | --- |
|  | **Donor** | **Before G-CSF mobilization**  **cell count (10^9^/liter)** | | | |  | **After G-CSF mobilization**  **cell count (10^9^/liter)** | | | | **Times of**  **leukapheresis** | **Components of the G-MNCs collected by leukapheresis (%)** | | | | | | |  |
|  |  | White-cell | Neutrophil | Lymphocyte | Monocyte |  | White-cell | Neutrophil | Lymphocyte | Monocyte |  | CD34^+^ | CD14^+^CD45^+^ | CD3^+^CD4^+^ | CD3^+^CD8^+^ | CD3^-^CD19^+^ | CD3^-^CD16^+^CD56^+^ | CD4^+^CD25^+^CD127^-^ |  |
|  | 01-W donor | 6.3 | 3.24 | 2.39 | 0.49 |  | 43.29 | 36.81 | 2.165 | 3.03 | First  Second | 0.48  0.38 | 27.5  37.9 | 17.2  13.7 | 11.8  8.7 | 4.7  3.5 | 4.9  4.6 | 1.0  0.7 |  |
|  | 02-Z  donor 1 | 4.02 | 2.19 | 1.6 | 0.19 |  | 42.15 | 35.83 | 2.72 | 3.03 | First  Second | 0.35  0.94 | 20.0  28.4 | 3.9  14.0 | 2.4  11.5 | 0.6  4.1 | 2.7  11.5 | 0.4  0.7 |  |
|  | 02-Z  donor 2 | 6.42 | 4.48 | 1.54 | 0.3 |  | 32.41 | 28.18 | 2.41 | 1.46 | First | 1.55 | 32.0 | 15.2 | 14.3 | 6.1 | 7.1 | 0.71 |  |
|  | 03-G  donor | 4.26 | 2.33 | 1.65 | 0.2 |  | 50.93 | 45.73 | 2.87 | 2.11 | First  Second | 0.11  0.13 | 21.88  26.74 | 27.04  22.63 | 9.62  7.38 | 9.15  8.43 | 3.72  12.99 | 1.05  1.13 |  |
|  | 04-D  donor | 7.51 | 5.84 | 1.28 | 0.33 |  | 56.11 | 49.38 | 5.05 | 1.68 | First  Second | 0.4  0.28 | 11.94  15.99 | 14.87  15.02 | 7.03  7.66 | 5.75  5.06 | 9.23  8.84 | 1.11  0.91 |  |
|  | 05-Z donor | 3.77 | 2.02 | 1.65 | 0.04 |  | 35.05 | 30.27 | 3.45 | 1.98 | First | 0.19 | 17.84 | 9.9 | 9.46 | 8.15 | 8.65 | 0.85 |  |
|  | 06-L donor | 6.91 | 4.03 | 2.4 | 0.36 |  | 44.41 | 38.18 | 3.33 | 2.29 | First | 1.12 | 16.47 | 10.67 | 2.08 | 3.77 | 3.57 | 0.21 |  |
|  | 07-X donor | 7.5 | 3.91 | 2.97 | 0.32 |  | 69.07 | 58.32 | 7.48 | 2.26 | First | 0.02 | 5.52 | 18.42 | 10.82 | 4.55 | 7.77 | 1.11 |  |
|  | 08-D donor | 7.49 | 4.66 | 2.31 | 0.33 |  | 34.16 | 28.15 | 4.16 | 1.56 | First | 0.07 | 15.17 | 10.17 | 13.2 | 5.98 | 3.28 | 0.97 |  |
|  | 09-L donor | 5.97 | 3.6 | 1.92 | 0.3 |  | 43.77 | 38.96 | 2.63 | 2.19 | First | 0.23 | 8.48 | 8.52 | 6.89 | 2.47 | 5.75 | 0.8 |  |
|  | 10-L donor | 5.65 | 3.99 | 1.24 | 0.39 |  | 39.09 | 34.11 | 3.07 | 1.61 | First | 0.14 | 9.06 | 20.35 | 8.83 | 2.85 | 5.96 | 1.0 |  |
|  | 11-S donor | 3.94 | 2.2 | 1.5 | 0.2 |  | 37.96 | 32.58 | 3.36 | 1.81 | First  Second | 0.07  0.3 | 15.21  10.25 | 1.83  12.57 | 16.91  15.94 | 5.68  6.7 | 26.65  4.51 | 0.5  3.07 |  |
|  | 12-L donor | 5.04 | 3.21 | 1.45 | 0.27 |  | 41.69 | 35.85 | 3.54 | 2.29 | First  Second | 0.22  0.49 | 16.27  23.55 | 7.52  11.88 | 13.9  15.10 | 2.11  7.13 | 2.09  2.57 | 1.4  0.82 |  |
|  | *G-CSF* granulocyte colony-stimulating factor | | | | | | | | | | | | | | | | | |  |

**Table S 2.** Details of blood cell counts before G-CSF mobilization and at leukopheresis, and the components of the G-MNCs collected by leukopheresis in donors.

G-MNCs were collected using a cell separator (COM.TEC, Fresenius Kabi) according to the stem cell collection protocol, and the proportion of different immunocytes was determined by FACS analysis. Based on the amount of immunocyte and platelet, leukapheresis was performed twice in six donors and only once in another six donors. G-CSF, granulocyte colony-stimulating factor.


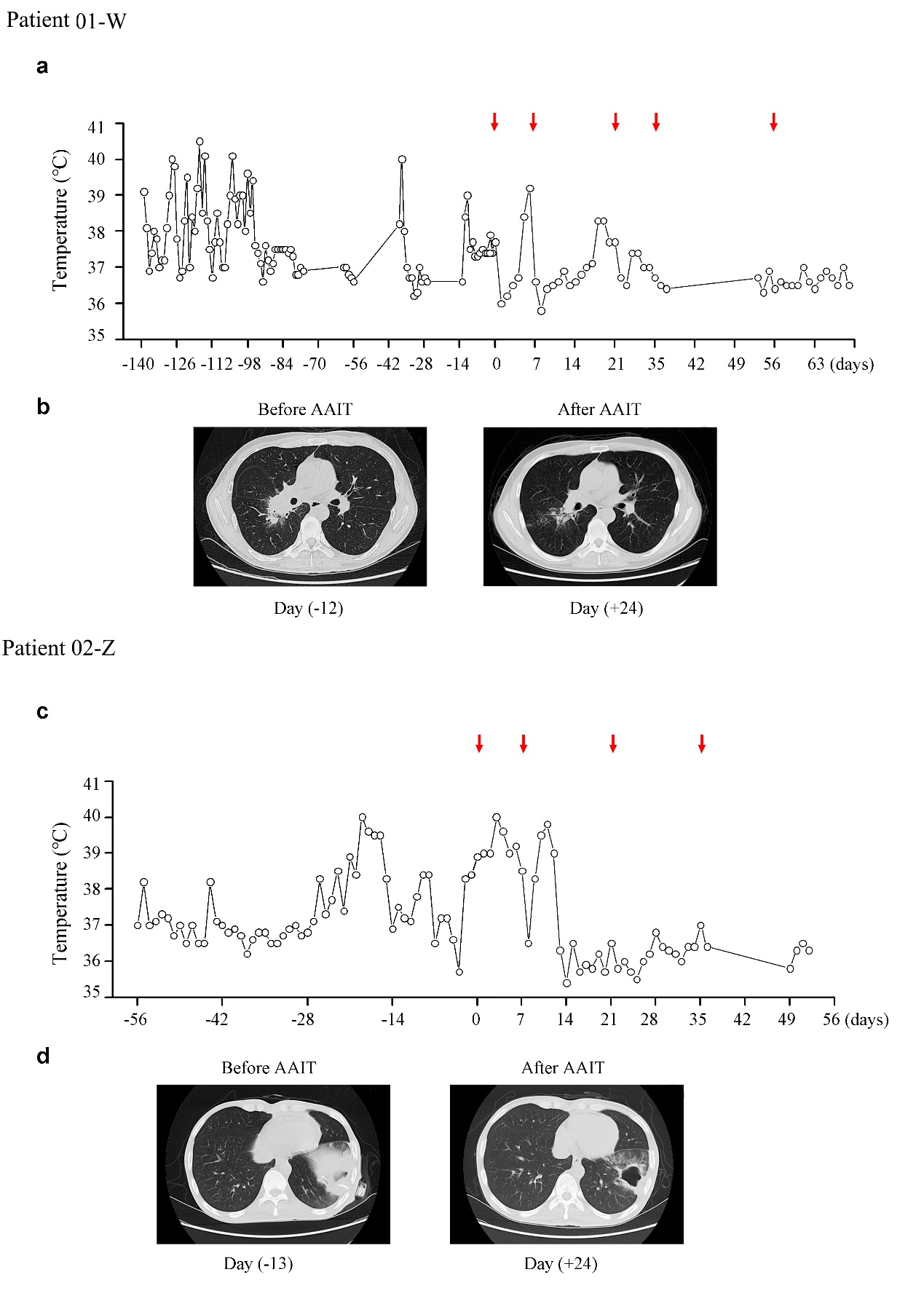


**Fig S 1.** Effects of AAIT on clinical symptoms in severely immunosuppressed AIDS patients.

Representative effects of AAIT on clinical symptoms and opportunistic infections (OIs) are shown in patient 1 (02-Z, **a** and **b**) and patient 2 (01-W, **c** and **d**). The therapeutic protocol is noted along the top of the figure; the arrows represent the time of G-MNCs transfusion.


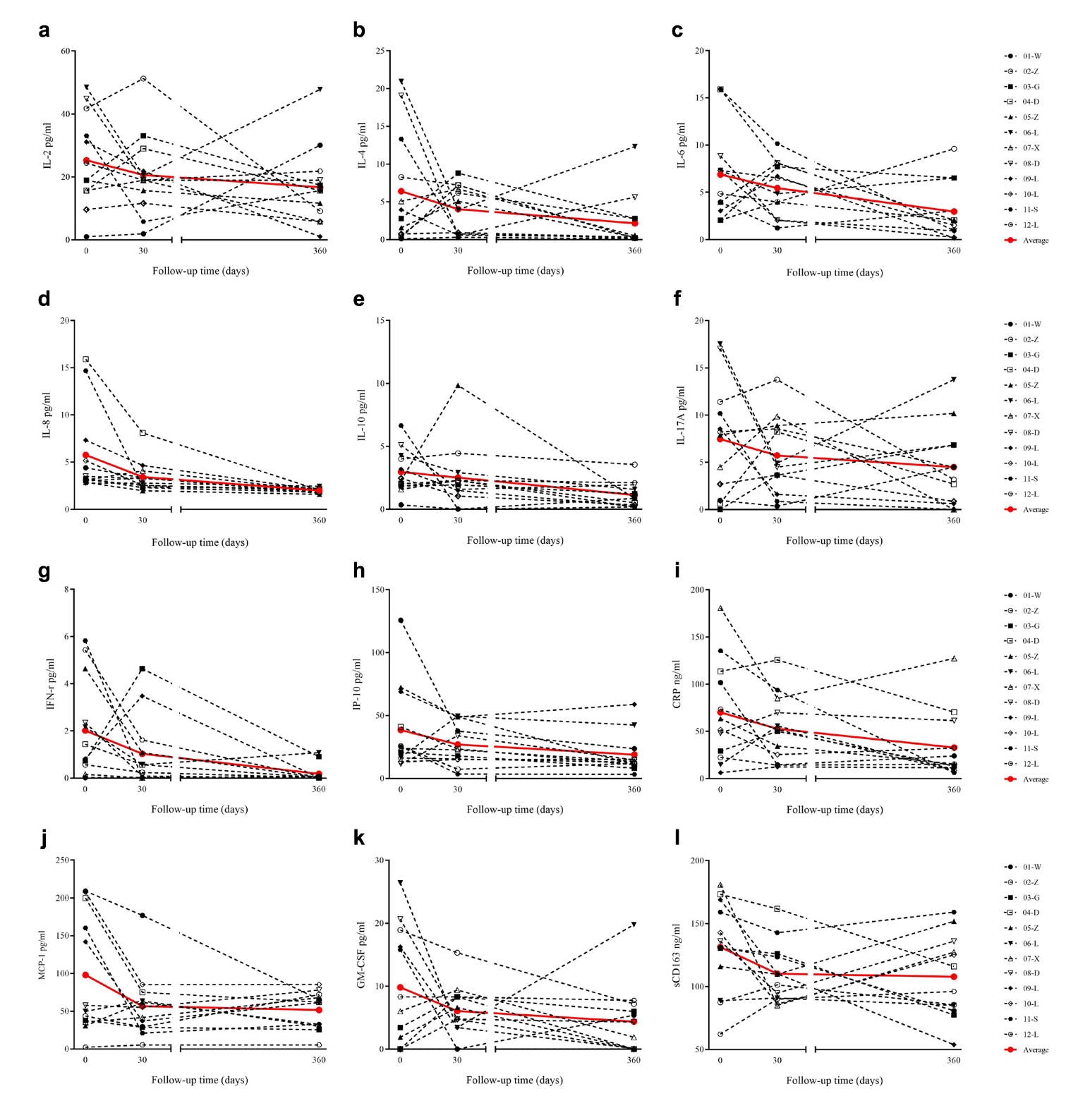

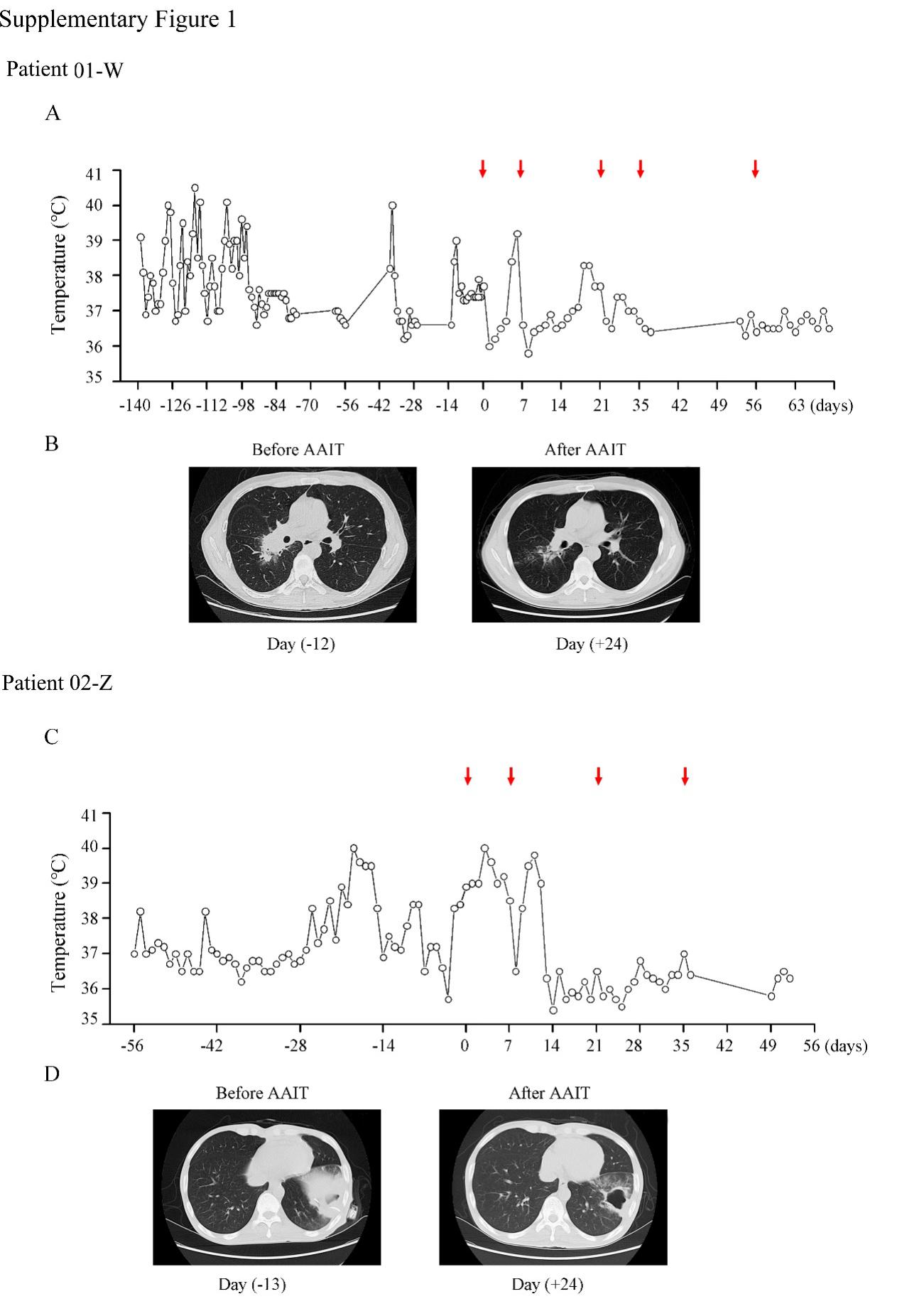


**Fig S 2.** Serum cytokine levels within 12 months after AAIT in patients.

Serum cytokines were screened within 30 days and 360 days after AAIT. **a-l** Serum levels of IL-2, IL-4, IL-6, IL-8, IL-10, IL-17A, IFN-r, IP-10, CRP, MCP-1, GM-CSF, and sCD163. Red color line represents the average level of each cytokine. IL-2, Interleukin-2; IL-4, Interleukin-4; IL-6, Interleukin-6; IL-8, Interleukin-8; IL-10, Interleukin-10; IL-17A, Interleukin-17A; IFN-r, Interferon r; IP-10, Interferon-inducible protein-10; CRP, C reactive protein; MCP-1, Monocyte chemotatic protein-1; GM-CSF, Granulocyte-macrophage colony-stimulating factor; sCD163, Soluble cluster of differentiation 163.

**
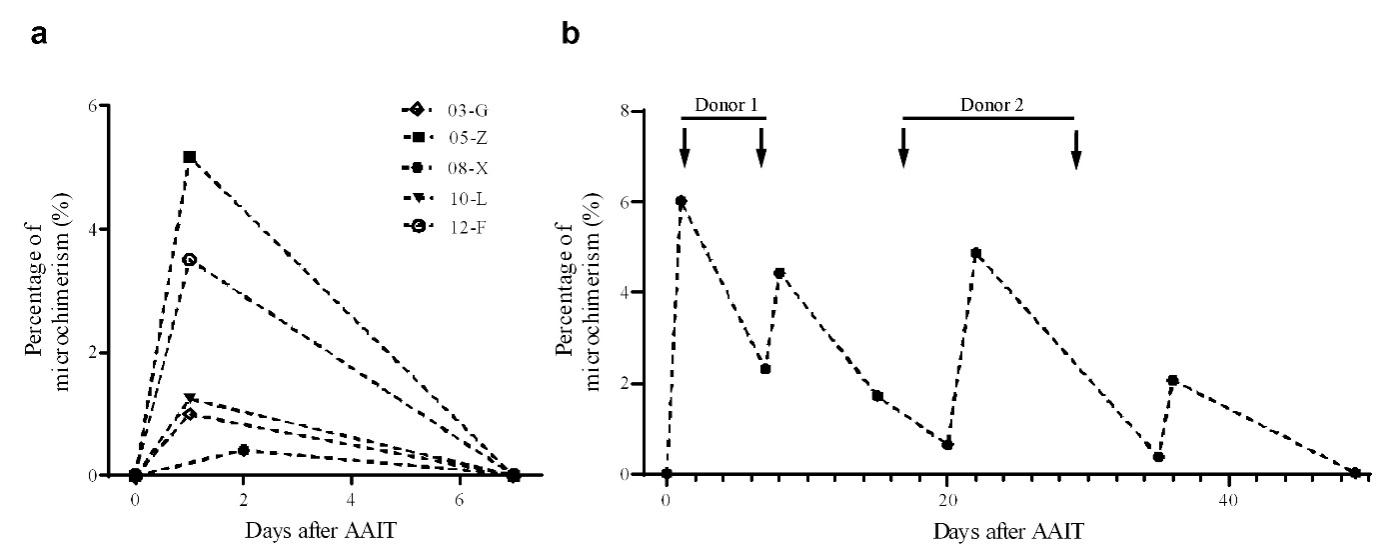
**

**Fig S 3.** Dynamics of donor cell microchimerism following AAIT treatment.

Microchimerism was respectively recorded in HLA-mismatched and HLA-matched recipients. **a** Microchimerism at day 1 and day 7 was recorded in patients 03-G, 05-Z, 10-L, and 12-F after AAIT treatment. Microchimerism at day 2 and day 7 was recorded in patient 08-X after AAIT treatment. **b** Dynamics of microchimerism was recorded in patient 02-Z, who received both HLA-matched (donor 1) and HLV-mismatched (donor 2) G-MNCs transfusions. Arrows represent the time of G-MNCs transfusion.

|  | **Table S 3.** ART regimens for 12 severely immunosuppressed AIDS patients. | | | |  |
| --- | --- | --- | --- | --- | --- |
|  | **Patient** | **ART regimen before AAIT** | **ART regimen during or**  **after AAIT** | **Reasons for regimen switch** |  |
|  | 01-W | 3TC + TDF + EFV | 3TC + TDF + LPV/r | Intolerance to EFV |  |
|  | 02-Z | 3TC + TDF + EFV | 3TC + AZT + DTG | Drug resistance to 3TC, TDF and EFV |  |
|  | 03-G | 3TC + TDF + LPV/r | 3TC + TDF + LPV/r | No switch |  |
|  | 04-D | 3TC + AZT + LPV/r | 3TC + AZT + LPV/r | No switch |  |
|  | 05-Z | 3TC + TDF + LPV/r | 3TC + TDF + LPV/r | No switch |  |
|  | 06-L | 3TC + TDF + EFV | 3TC + AZT + LPV/r | Drug resistance to TDF and EFV |  |
|  | 07-X | 3TC + TDF + EFV | 3TC + TDF + EFV | No switch |  |
|  | 08-D | 3TC + TDF + EFV | 3TC + TDF + NVP | Intolerance to EFV |  |
|  | 09-L | 3TC + TDF + EFV | 3TC + TDF + EFV | No switch |  |
|  | 10-L | 3TC + TDF + EFV | 3TC + TDF + EFV | No switch |  |
|  | 11-S | 3TC + TDF+EFV | 3TC + TDF + EFV | No switch |  |
|  | 12-L | 3TC + TDF + EFV | 3TC + TDF + EFV | No switch |  |
|  | *3TC* lamivudine, *TDF* tenofovir, *EFV* efavirenz, *LPV/r* lopinavir/ritonavir, *DTG* dolutegravir, *AZT* zidovudine, *NVP* nevirapine. | | | |  |

**Table S 3.** ART regimens for 12 severely immunosuppressed AIDS patients.

The ART regimen before AAIT and after AAIT were recorded for the 12 patients, four patients have changed ART drug, the reason for patients 02-Z and 06-L is a result of drug resistance, the reason for patient 01-W and 08-D is due to intolerance to EFV. 3TC, lamivudine; TDF, tenofovir; EFV, efavirenz; DTG, dolutegravir; LPV/r, lopinavir/ritonavir; AZT, zidovudine; NVP, nevirapine; RAL, raltegravir.
